# Coming to terms with climate change: a glossary for climate change impacts on mental health and well-being

**DOI:** 10.1136/jech-2024-222716

**Published:** 2024-12-18

**Authors:** Claire L Niedzwiedz, Jonathan R Olsen, Jala Rizeq, Tsion Afework, Chiara K V Hill-Harding, Richard J Shaw, Rhian Thomas, Symon M Kariuki, Srinivasa Vittal Katikireddi, Andrew J Weaver, Gina Martin, Hester Parr, Esther K Papies

**Affiliations:** 1 School of Health and Wellbeing, University of Glasgow, Glasgow, UK; 2 MRC/CSO Social and Public Health Sciences Unit, University of Glasgow, Glasgow, UK; 3 College of Social Sciences, University of Glasgow, Glasgow, UK; 4 School of Psychology and Neuroscience, University of Glasgow, Glasgow, UK; 5 School of Geographical and Earth Sciences, University of Glasgow, Glasgow, UK; 6 KEMRI-Wellcome Trust Research Programme, Kilifi, Kenya; 7 School of Earth and Ocean Sciences, University of Victoria, Victoria, British Columbia, Canada; 8 Faculty of Health Disciplines, Athabasca University, Athabasca, Alberta, Canada; 9 Radboud Universiteit, Nijmegen, Netherlands

**Keywords:** MENTAL HEALTH, ENVIRONMENTAL HEALTH, CLIMATE CHANGE, GEOGRAPHY, PSYCHOLOGY

## Abstract

Climate change is a major threat to global health. Its effects on physical health are increasingly recognised, but mental health impacts have received less attention. The mental health effects of climate change can be direct (resulting from personal exposure to acute and chronic climatic changes), indirect (via the impact on various socioeconomic, political and environmental determinants of mental health) and overarching (via knowledge, education and awareness of climate change). These impacts are unequally distributed according to long-standing structural inequities which are exacerbated by climate change. We outline key concepts and pathways through which climate change may affect mental health and explore the responses to climate change at different levels, from emotions to politics, to highlight the need for multilevel action. We provide a broad reference to help guide researchers, practitioners and policy-makers in the use and understanding of different terms in this rapidly growing interdisciplinary field.

**WHAT IS ALREADY KNOWN ON THIS SUBJECT**

- Climate change can have direct, indirect and overarching effects on mental health.
- Climate change may impact mental health via various socioeconomic, environmental and political pathways, exacerbating existing inequities.
- There is a lack of clarity around key terminology, such as climate change worry and anxiety.

**WHAT THIS STUDY ADDS**

- Key concepts relating to climate change and mental health are discussed and clarified.
- Individual, community, organisational and political pathways through which climate change may impact mental health are outlined.
- Key empirical evidence and priority areas for future research are identified.

## Introduction

**Figure 1:**
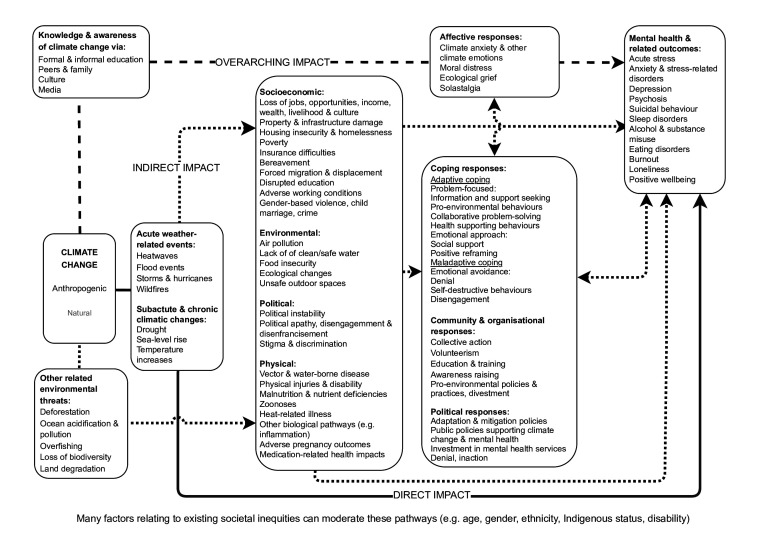
Pathways between climate change and mental health.

 Climate change is one of the biggest challenges to public health. As well as impacting physical health, it is increasingly recognised that people’s mental health is adversely affected, with disadvantaged groups and people from low-income and middle-income countries particularly at risk.^1^ Evidence on climate change-related mental health effects is rapidly increasing, but health and social systems are currently poorly equipped to address this demand.^2^ Terminology used to describe mental health is important to avoid pathologising, stigmatising or trivialising the mental health implications of climate change.^1^ Building on a previous glossary about climate change for public health practice,^3^ this article outlines and discusses key concepts related to climate change and mental health, drawing on a range of existing approaches and frameworks.^12 4^,^8^ It aims to improve understanding of the mental health implications of climate change and emerging interdisciplinary research in this space. Climate change may affect mental health through various complex pathways (figure 1). Impacts can be classified as direct, indirect and overarching,^4 5^ although the distinction can be blurred and evidence for some pathways is mixed.

## Direct impacts of climate change on mental health

Direct impacts stem from the personal experience of acute weather-related events, as well as subacute and chronic hazards arising from longer-term climatic changes. Direct impacts of climate change often disproportionately affect disadvantaged groups,^1^ due to factors such as age, gender, ethnicity, socioeconomic position, geographical setting, disability and pre-existing physical and mental health conditions. Different factors can interact to exacerbate mental health inequalities. For example, people living in low-income and middle-income countries, particularly within remote or rural island communities, are disproportionately impacted by climate change while also often being among those with the least resources to adapt to and mitigate its effects.^9^

### Acute weather-related events

Sudden-onset extreme weather events and hazards affecting mental health include heatwaves, wildfires, storms, hurricanes and floods, now increasing both in frequency and intensity. Experiencing these events may have acute effects on mental health, potentially triggering acute stress reactions, adjustment disorder and post-traumatic stress disorder (PTSD).^10^,^13^ Extreme weather events can also impact sleep, causing insomnia and nightmares, and possibly contributing to the onset of depression and PTSD.^14^ Mental health impacts of severe weather-related events may also have long-term implications. Concerns about future flooding can adversely affect mental health, with anxiety tending to peak when adverse weather is forecast,^15^ or during periods of heavy rainfall among people with prior exposure to flood events.^16 17^ Heatwaves may increase suicidal behaviour, hospitalisations for mental illness and adversely impact community mental health and well-being.^18^ Similarly, exposure to wildfire smoke has been linked with increased emergency department visits for anxiety disorders.^19^

### Subacute climatic changes

Subacute climatic changes include slower-onset and less visible effects, such as increasing ambient outdoor temperatures, prolonged drought and excessive rainfall.^18 20 21^ Drought can vary in terms of duration and intensity and these characteristics may relate to differing mental health impacts for distinct areas.^22^ Links between periods of severe drought and increased psychological distress have been identified, especially in rural areas,^22 23^ and among farmers who experienced high levels of suicidal ideation.^24^ Variability in long-term local temperatures has also been linked with suicide and other outcomes including psychological distress and cognitive difficulties.^25^

### Chronic climatic changes

Observing sea-level rise and gradual changes in the environment, such as melting sea ice, land degradation and loss of biodiversity can be considered chronic exposures resulting from climate change.^4 5 25^ Increases in sea level can have major impacts on individual and community mental health among those experiencing the worst effects, such as small island states and coastal communities.^26 27^ Directly experiencing and being forced to adapt to sea level increases over a long period of time is already leading to extreme and disabling psychological distress in countries like Tuvalu, particularly among low-income households.^28^

## Indirect impacts of climate change on mental health

Indirect impacts, arising from extreme weather events or chronic climatic changes, can operate via deteriorating socioeconomic, political and environmental conditions which influence mental health.^1 29^ Indirect effects may also operate via physical health (eg, heat-related illness) but these are not discussed in detail. Many of these factors can interact or accumulate to exacerbate health inequalities and pre-existing mental health conditions. For example, at the individual level, people living with mental illness may be more likely to have lower income, be exposed to poorer working conditions (eg, outdoor working during extreme heat) and live with multiple health conditions. At the community level, they may also be more likely to live in disadvantaged neighbourhoods with higher levels of air pollution.^30 31^

### Socioeconomic pathways

Socioeconomic pathways through which climate change can affect mental health are numerous and exacerbated by existing disparities in wealth and other resources.^1^ Climate change has major implications for jobs and livelihoods, on which mental health is often dependent. This particularly affects farmers, fisher and Indigenous peoples, who are closely connected to, and sometimes reliant on the land and sea for their livelihoods. Damage to crops and livestock may result in loss of income and wealth, leading to or exacerbating poverty. Extreme weather can destroy homes, property and other infrastructure, leading to secondary stressors such as displacement, housing insecurity and homelessness, which are all known to impact mental health. Difficulties in obtaining insurance in flood-prone areas can result in financial difficulties, especially after repeated events, adding to stress and anxiety.^17^ Occupational conditions, particularly among outdoor workers, are deteriorating due to higher temperatures and other climate-related exposures, potentially leading to increased stress, anxiety and burn-out. Higher temperatures are associated with increases in conflict and aggressive behaviour, resulting in disrupted social relationships, gender-based violence and criminal behaviour, including physical and sexual assaults,^32^ with disproportionate effects on women and girls.^33 34^ Children and young people are unfairly impacted via disruptions to education,^35^ which is a key social determinant of mental health.^29^ Heat can negatively impact cognitive function and hence, academic performance, and acute events such as storms, wildfires and floods can adversely affect school attendance through a number of pathways, such as school closures and malnutrition.^35^ Rising sea levels, extreme heat and drought are leading to forced migration as specific inhabited areas become less liveable or vanish completely, leading to loss of autonomy and increasing risks of mental health disorders. The loss of places and culture that are important to people’s sense of self and identity can also lead to feelings of loneliness and isolation.

### Environmental pathways

Climate change affects various environmental determinants of mental health. Air pollution and climate change are deeply interlinked; both caused by the extraction and use of fossil fuels and industrial agriculture. Increasing temperatures and drier conditions exacerbate general levels of air pollution; and heatwaves can lead to wildfires which emit harmful greenhouse gases and particulate matter (PM).^36^ Air pollution is linked to increased symptoms of depression and anxiety, with some studies demonstrating that O_3_ (ozone) is related to a short-term impact on depression among young people in particular, with fine particulate matter (PM_2.5_) and nitrogen dioxide suggested to have longer-term impacts.^37^ Potential neurobiological mechanisms include oxidative stress, neuroinflammation, most of which can result in changes to frontolimbic brain regions.^38^ Children, expectant mothers and older people are particularly affected by extreme temperatures, air pollution and other environmental hazards, in addition to these restricting their opportunities to be outdoors and increasing their risk of social isolation.

Climate change influences access to clean and safe drinking water and the four key components of food security: availability, access, utilisation and stability.^39^ Loss of biodiversity can lead to land degradation, when climate change alters soil quality and quantity that affects agricultural yield.^40^ In low-income coastal communities, marine resources are vital for animal protein and nutrition. Climate change contributes to deterioration of marine resources, worsening already-present problems, such as human-induced overfishing and ocean pollution.^41^ This deterioration of marine resource management may also contribute to loss of biodiversity and food insecurity at the same time.^42^ Food insecurity not only influences nutrition but can also impact mental health,^43^ via stress and anxiety around the ability to acquire food, which contributes to feelings of shame and guilt.^43 44^ The impact of climate change on eating disorders is also an area of concern.^45^

### Political pathways

The political determinants of mental health are undermined by climate change. Forced migration and displacement resulting from climate change can increase political instability, leading to effects on democracy and the economy. Disenfranchisement of displaced communities may arise, leading to stigmatisation and discrimination of marginalised groups, adversely impacting mental health. Inadequate responses to climate change by governments, corporations and other institutions can result in political apathy and disengagement, further compounded by extreme inequality. Disillusionment and disengagement with electoral politics and climate change governance among young people is a potentially growing issue,^46 47^ with many experiencing feelings of betrayal around government responses to climate change.^48^

## Overarching impacts of climate change on mental health

### Knowledge and awareness

There is growing recognition that knowledge, awareness and anticipation of the effects of climate change may impact mental health.^4^ This can affect people who are not directly affected by climate change. Abstract knowledge of climate change may be as important for mental health as observing local changes in the environment, among communities worst affected.^28^ Factors shaping knowledge include formal and informal education, peer and family relationships, culture and media.^7^ School and education systems can influence the framing of climate change and provide accurate knowledge to foster a sense of empowerment and encourage positive action, enhancing well-being. If the issue is inadequately addressed in educational institutions (including an abundance of negative messaging), young people may not develop adequate knowledge of climate change, or how to cope with its implications, leading to negative emotions like powerlessness, helplessness and sadness.^49^ Social relationships with peers and family members can contribute to people’s understanding and attitudes towards climate change, with conflicting views leading to feelings of frustration and isolation. The community and culture in which an individual lives can also contribute, which may have positive or negative effects, depending on general attitudes and beliefs about climate change, as well as resources for and engagement in collective action.^7^ The media play a key role in communicating information about climate change, shaping knowledge and attitudes.^50^ Media can increase motivation to positively engage with climate change.^51 52^ However, sensationalist news and digital media coverage can sometimes cause fear and anxiety especially in the absence of solutions.^52^

## Responses to climate change

Understanding how people, communities and institutions respond to climate change is crucial to tackling the challenges it presents to mental health. Responses to climate change are influenced by values, beliefs, group affiliations and identity,^6^ as well as social, cultural, political and geographic factors.^53^

### Affective responses

#### Climate emotions

Many people experience a range of complex emotions in relation to the climate crisis, referred to as ‘*climate emotions*’, ranging from despair and grief, to optimism and empowerment.^4854^,^56^ These can relate to wider ‘ecological’ or ‘Earth’ emotions concerning environmental crises, such as biodiversity loss and species extinction.^57^ Responding to changing emotional responses to environmental crises, the environmental philosopher Albrecht introduced the term *psychoterratic syndromes* to mean ‘earth-related mental illness where people’s mental well-being (psyche) is threatened by the severing of ‘healthy’ links between themselves and their home/territory’.^58^ However, it is possibly unhelpful to refer to mental illness in this context as this risks pathologising appropriate emotional reactions. Albrecht defined a new form of ‘psychoterratic illness’ which he termed *solastalgia*. Solastalgia is defined as ‘the pain or distress caused by the loss of, or inability to derive, solace connected to the negatively perceived state of one’s home environment’.^58^ It has also been described as ‘homesickness’ felt when still living at home, arising from feeling disconnected and a profound sense of loss towards the environment and land once known.^58 59^ Solastalgia is related to *ecological grief*, which is defined as ‘grief associated with physical ecological losses, such as the physical disappearance, degradation and/or death of species, ecosystems and landscapes, driven by climate change’.^60^

#### Climate worry

Worry and concern reflect negative or apprehensive thoughts and emotional experiences due to preoccupation with possible future threats or negative expectations.^61 62^ Although characterised as a negative cognitive-emotional experience, worry can have motivational impacts that allow people to respond to and prepare for potential threat.^63 64^ Within the context of the climate crisis, *climate worry* is characterised by verbal-linguistic thoughts (rather than images) about the changes that are taking place, or may occur, in the climate system and the possible implications of these changes.^65^ Importantly, climate worry has been related to engagement in proenvironmental behaviour.^66 67^ However, when worry becomes difficult to manage, persistent and repetitive, it can be an indicator of climate anxiety.^62 65^

#### Climate and eco-anxiety

Emotional responses to the climate crisis and its anticipated impacts have been conceptualised under the umbrella term *climate anxiety* to capture both adaptive and maladaptive (or pathological) functions. Anxiety is characterised by negative emotionality, accompanied by somatic or physical symptoms and worry about the future that varies in degree of severity and function; it can be experienced as adaptive precautions for potential threats or maladaptive dysregulation of emotion.^68^,^70^ Climate anxiety can be considered a component of the broader term *eco-anxiety*. Eco-anxiety is the experience of challenging emotions relating to environmental issues, not necessarily specific to climate change. Climate anxiety is considered a justifiable and healthy response to the climate crisis.^71^ However, without appropriate coping mechanisms and resources, a clinical presentation of anxiety may be apparent when anxiety and worries about the past, present and/or future, are difficult to manage and impact on an individual’s daily functioning.^72^ Qualitative interviews with people experiencing climate anxiety contain accounts of panic attacks, rumination, irritability, loss of appetite, concentration and sleep,^62 73 74^ consistent with physical and cognitive symptoms of anxiety. Interventions (individual level and community level) will likely be needed for people (particularly those with pre-existing mental disorders) who experience heightened and sometimes debilitating levels of distress,^71^ but a focus on prevention is first needed to enable people to manage difficult emotions and channel them into positive action. Several critiques of the terms climate and eco-anxiety have been articulated. These highlight that discussions around climate anxiety often centre white and privileged experiences, which risks ignoring the voices of those most impacted by the climate crisis.^75^ Others emphasise that the focus on eco-anxiety has led to a medicalised perspective which places too much emphasis on individuals and depoliticises the Anthropocene.^76 77^

#### Environmental moral distress

In response to critiques around eco-anxiety, the concept of *environmental moral distress* has been proposed to help centre ecological emotions within their social, cultural and political contexts.^77^ Environmental moral distress refers to the anguish people experience in response to ecological crises, including climate change. Moral distress results from experiencing a moral event (a moral dilemma or uncertainty, such as reneging on climate policy) which causes psychological distress.^77^ Moral injury is a type of trauma proposed to result from sustained moral distress and can be a precursor to PTSD,^77^ characterised by guilt, existential crisis and loss of trust that may develop following a perceived moral violation.^6 78^ The violation can occur when one acts against one’s sense of what is right (referred to as moral injury-self), or when an outside actor in a position of authority (eg, an institution or political leader) betrays their values (moral injury-other).^6^ Experiences of moral injury are highly influenced by people’s social context and geographical location.^6 77^ For example, in high-income countries, acquiring material possessions and wealth beyond what is necessary and knowing that this has contributed to climate change may be a source of moral injury. Whereas moral injury due to the actions of others may be more likely to occur among those directly and severely impacted by climate change, such as Indigenous communities, when considering how high-income countries have exploited local environments in lower-income countries for their own profit.^6^

#### Climate trauma

*Climate trauma* refers to psychological distress which results from experiencing a damaging and life-threatening climate-related event. It is thought that neurocognitive mechanisms involved may resemble those found in individuals with PTSD, manifesting as frontal hyperarousal and deficits in processing efficiency.^79^ The term ‘pre-traumatic stress syndrome’ has been suggested as involving distress about an anticipated future, rather than a past event.^80^ However, these concepts have mainly been discussed in the humanities and social sciences, perhaps leading to overmedicalisation. Others have conceptualised climate trauma through the lens of cultural trauma, not just inducing trauma under specific circumstances, but an ‘ever-present, ever-growing threat to the biosphere, one that calls into question our shared identity: What does it mean to be ‘‘human’’ in the Anthropocene?’.^81^ Climate trauma is theorised to explain our collective paralysis in response to the threat of climate change, which can lead to distress and depression.

#### Coping responses

To manage climate emotions adaptive and maladaptive coping responses may be evident.^82^ Coping responses can be affective, cognitive and behavioural.^70^ Adaptive coping may be problem-focused, encompassing strategies such as active coping, information seeking, planning and collaborative problem-solving. Emotional approach coping strategies include social support, positive reframing, acceptance and compassion. Maladaptive coping covers emotional avoidance coping strategies such as denial, behavioural disengagement, blame-shifting, substance use and other self-destructive behaviours.^70 82^ The mental health impacts of different coping responses and strategies in relation to climate change require additional research. Engaging in individual mitigation strategies, such as adopting eco-friendly behaviours, has been positively correlated with well-being outcomes.^83^ The potentially bidirectional relationship between climate anxiety and proenvironmental behaviours remains unclear and is likely influenced by additional aspects, such as emotions and coping strategies, temporal dynamics, and other sociodemographic and geographical factors.^84^ Engagement in climate action may promote well-being and help manage climate anxiety, but there are risks of *burn-out*—overwhelming stress accumulated over time via exposure to emotionally demanding situations.^85^

#### Community responses

Climate anxiety and concern can have positive community benefits via increased constructive civic engagement and action, including canvassing, personal and group efficacy to solve community issues, and voting behaviour.^86^ Communities can also be positively impacted following extreme weather events, for example, volunteerism and collective activities in response to flooding disasters have been found to provide some mitigation against impacts on mental health and wellbeing,^17 87^ and contribute to renewed group identity and sense of community.^8^ This has been termed ‘communal coping’ and describes the process of communities uniting to become cohesive groups who view uncertainty and worry as solvable via collective action.^87^ However, negative responses may also arise if community resources are overwhelmed or where there are low levels of social cohesion.

#### Organisational responses

Organisations within public, private and third sectors, such as healthcare settings, schools and universities, play an important role in climate change mitigation and adaptation, and hence in factors that affect climate change-related mental health.^88^ Organisations can reduce emissions through updating their own practices, such as buildings or supply chains, but also shape decisions available to citizens, social norms and policy through links to political decision-makers.^89^ Health professionals should be trained in sustainability and climate literacy, and greater emphasis and investment in the prevention of mental disorders is required. Schools and universities have opportunities to educate others on climate change mitigation and adaptation, and on the links between climate change and mental health. School curricula that positively address the issue of climate change and engage learners early in sustainable practices and positive coping mechanisms can help create a generation that is aware and engaged in climate change mitigation. All of these responses have the potential to affect the mental health of students, staff and citizens. For example, a recent study at a large UK university demonstrated that students’ beliefs about the university’s climate actions and the perception of their concerns being dismissed positively correlated with students’ climate anxiety.^49^ Students also called for more climate change-related teaching and mental health support.

#### Political responses

Climate change governance can be enacted at various scales including international, national and local, with varying levels of ambition and success. The impacts on mental health and well-being are not well researched, but many young people feel disillusioned with governmental responses to climate change.^48^ Youth activists have expressed their disillusionment with the Conference of the Parties (COP) process which they feel exacerbates colonial power imbalances, marginalises Indigenous peoples and promotes intersectional exclusion, where minority groups are made to feel unwelcome, and their voices disregarded.^47^ Public policies that help to rebalance the social and economic determinants of mental health are urgently needed to help prevent climate change-related mental health problems. This may include reducing poverty, ensuring access to secure housing and investing in sustainable food systems. Investment in mental health services and climate-resilient health infrastructure is also needed, as well as funding for climate change and mental health research.

## Concluding comments

In this article, we have outlined key terms relating to climate change and mental health as well as the potential pathways and responses involved, many of which require additional research. The choice of content is influenced by our own backgrounds, experiences and disciplinary perspectives. Different cultures and languages likely have their own unique terms to describe climate change and mental health experiences. There is an urgent need for more research from countries most affected by the climate crisis particularly within the Global South and for longitudinal studies to track changes over time. Interdisciplinary research is necessary to fully understand the mechanisms through which climate change affects mental health, as well as how mitigation strategies and responses at multiple levels may impact mental health in different contexts.
